# Empagliflozin Preserves Skeletal Muscle Function in a HFpEF Rat Model

**DOI:** 10.3390/ijms231910989

**Published:** 2022-09-20

**Authors:** Ephraim B. Winzer, Antje Schauer, Erik Langner, Antje Augstein, Keita Goto, Anita Männel, Peggy Barthel, Anett Jannasch, Siegfried Labeit, Norman Mangner, Axel Linke, Volker Adams

**Affiliations:** 1 Laboratory of Molecular and Experimental Cardiology, TU Dresden, Heart Center Dresden, 01307 Dresden, Germany; 2 Department of Cardiac Surgery, TU Dresden, Heart Center Dresden, 01307 Dresden, Germany; 3 Medical Faculty Mannheim, University of Heidelberg, 69117 Heidelberg, Germany; 4 DZHK (German Center for Cardiovascular Research), Partner Site Heidelberg/Mannheim, 68167 Mannheim, Germany

**Keywords:** HFpEF, ZSF1, SGLT2 inhibitor, MuRF1, skeletal muscle dysfunction, muscle atrophy

## Abstract

Besides structural alterations in the myocardium, heart failure with preserved ejection fraction (HFpEF) is also associated with molecular and physiological alterations of the peripheral skeletal muscles (SKM) contributing to exercise intolerance often seen in HFpEF patients. Recently, the use of Sodium-Glucose-Transporter 2 inhibitors (SGLT2i) in clinical studies provided evidence for a significant reduction in the combined risk of cardiovascular death or hospitalization for HFpEF. The present study aimed to further elucidate the impact of Empagliflozin (Empa) on: (1) SKM function and metabolism and (2) mitochondrial function in an established HFpEF rat model. At the age of 24 weeks, obese ZSF1 rats were randomized either receiving standard care or Empa in the drinking water. ZSF1 lean animals served as healthy controls. After 8 weeks of treatment, echocardiography and SKM contractility were performed. Mitochondrial function was assessed in saponin skinned fibers and SKM tissue was snap frozen for molecular analyses. HFpEF was evident in the obese animals when compared to lean—increased E/é and preserved left ventricular ejection fraction. Empa treatment significantly improved E/é and resulted in improved SKM contractility with reduced intramuscular lipid content. Better mitochondrial function (mainly in complex IV) with only minor modulation of atrophy-related proteins was seen after Empa treatment. The results clearly documented a beneficial effect of Empa on SKM function in the present HFpEF model. These effects were accompanied by positive effects on mitochondrial function possibly modulating SKM function.

## 1. Introduction

Heart failure with preserved ejection fraction (HFpEF) is a complex syndrome affecting a substantial proportion of all heart failure patients and is associated with high morbidity and mortality rates [1]. HFpEF is frequently seen in patients suffering from co-morbidities associated with the metabolic syndrome, namely obesity, hypertension, diabetes mellitus and hyperlipidemia (for review see [2]), suggesting that HFpEF is a systemic disease including alterations of the myocardium. Besides structural alterations in the myocardium, HFpEF is also associated with endothelial dysfunction and myopathy of the peripheral skeletal muscles (SKM) [3,4]. SKM atrophy and myopathy include molecular alterations such as fat infiltration [5], reduced mitochondrial oxidative capacity [6], shift in fiber type composition [7], decreased anabolic factors such as IGF-1 [8] and a reduced capillary to fiber ratio [9] in the affected SKM. With regard to SKM myopathy, the activation of the ubiquitin proteasome system (UPS) is a central step in promoting muscle wasting in probably all chronic muscle wasting states [10,11], including SKM atrophy in HFpEF [12]. Atrophic stress is promoted by a family of E3 ligases including the muscle ring finger protein 1 (MuRF1) and muscle atrophy F-box protein (MafBx). These E3 ligases trigger the ubiquitinylation of contractile proteins thereby marking them for degradation via the UPS with subsequent loss of myofibrils.

In contrast to the wealth of proven therapies for heart failure with reduced ejection fraction (HFrEF), most efforts to improve morbidity and mortality in HFpEF have failed so far (for review see [13]). The recently published EMPEROR-Preserved trial using Empagliflozin (Empa), a sodium glucose cotransporter-2 inhibitor (SGLT2i), reported positive effects on the combined clinical endpoint of cardiovascular death or hospitalization for heart failure compared to placebo in patients with HFpEF [14]. SGLT2i were originally developed as anti-diabetic drugs since they inhibit the glucose reabsorption in the proximal tubule of the kidney. The underlying molecular mechanisms, which are responsible for the beneficial effects in heart failure patients with and without diabetes mellitus, have been incompletely investigated, especially as SGLT2 is not detectable in normal and failing myocardium [15]. A number of theories are discussed to explain the compelling benefits of SGLT2i in heart failure including increased energy metabolism, anti-inflammatory effects, decreased oxidative stress and increased autophagy and lysosomal degradation (for review see [16]).

Only a few studies in rodents have been performed to investigate the impact of SGLT2i on SKM. Feeding db/db mice for 8 weeks with luseogliflozin resulted in an increased cross-sectional area of the soleus muscle, probably due to a suppression of Foxo1 expression [17]. Analyzing the impact of Empa on muscle endurance in an LAD-ligation model of HFrEF, Nambu and colleagues documented a significant increase in muscle performance without any changes in muscle weight, probably related to a restoration of mitochondrial fatty acid oxidation [18]. Accumulating evidence from preclinical studies suggests a protective effect of SGLT2i on mitochondrial function also in other organs such as the kidney [19] and heart [20,21].

Since skeletal muscle alterations in HFpEF are associated with mitochondrial dysfunction and energetic deficiencies [6,22] and SGLT2i were found to modulate muscle performance via an improvement of mitochondrial bioenergetics, the present study thought to investigate the impact of Empa on skeletal muscle function in an established animal model of HFpEF (ZSF1 rat model [23,24]). In addition, mitochondrial function, metabolic parameters and markers for muscle atrophy were analyzed. Since the soleus muscle is an oxidative muscle and therefore depends more on oxidative phosphorylation for proper function, we decided to perform functional and molecular analysis primarily in the soleus muscle.

## 2. Results

### 2.1. Impact of Empaliflozin on Physiological and Echocardiographic Parameter

At the age of 24 weeks, when the ZSF1-obese animals had already a HFpEF phenotype [23,24,25], they were randomized into a placebo or treatment group (receiving Empa for 8 weeks). Comparison of ZSF1-obese and ZSF1-lean animals at week 24 already detected a significant increase in body weight (lean: 252 ± 2 g vs. obese: 477 ± 7 g; *p* < 0.0001), E/é (lean: 18.6 ± 0.8 vs. obese: 22.8 ± 0.6; *p* < 0.0001). In contrast, the left ventricular ejection fraction (lean: 69.7 ± 0.9% vs. obese: 71.7 ± 1.3%) was preserved and not significantly different between both groups. Based on the echocardiographic evaluation, the ZSF1-obese animals developed HFpEF before they were randomized into either the Empa treatment or the placebo group.

The daily dose of Empa the animals received via the drinking water was between 26 and 38 mg/kg. As expected, and has already been described in earlier studies [23,24,25], at the age of 32 weeks the obese animals exhibited a significantly increased body weight, heart weight, kidney weight and blood glucose concentration, whereas the lung wet/dry ratio was significantly reduced (Table 1). With respect to echocardiographic parameters the obese animals showed a preserved systolic function (LVEF and LVFS), whereas the diastolic function was significantly impaired (increased E/é and left ventricular end-diastolic pressure (LVEDP)) when compared to the ZSF1-lean control group (Table 1). Treating the ZSF1-obese animals with Empa for 8 weeks resulted in a significant reduction in blood glucose concentration, a significant elevation of excreted glucose via the urine and an improvement in echocardiographic-determined diastolic functional parameters E/é and LVEDP. Empa also significantly reduced body weight, heart weight and lung wet weight normalized to tibia length but had no effect on aortic blood pressure (Table 1). In summary, these data clearly show that Empa is effective in ZSF1-obese animals (reduction in blood glucose) and that it significantly improves diastolic function.

Assessment of food consumption in the three different groups over the treatment period of 8 weeks revealed that the lean animals consumed significant less food per day when compared to the obese or obese + Empa animals. No significant difference was observed between obese and obese + Empa (lean: 17.8 ± 0.5 g/d; obese: 25.4 ± 0.5 g/d; obese + Empa: 26.0 ± 0.7 g/d; *p* < 0.001 lean vs. obese or obese + Empa).

### 2.2. Impact of Empaliflozin on Muscle Trophicity and Function

**Figure 1:**
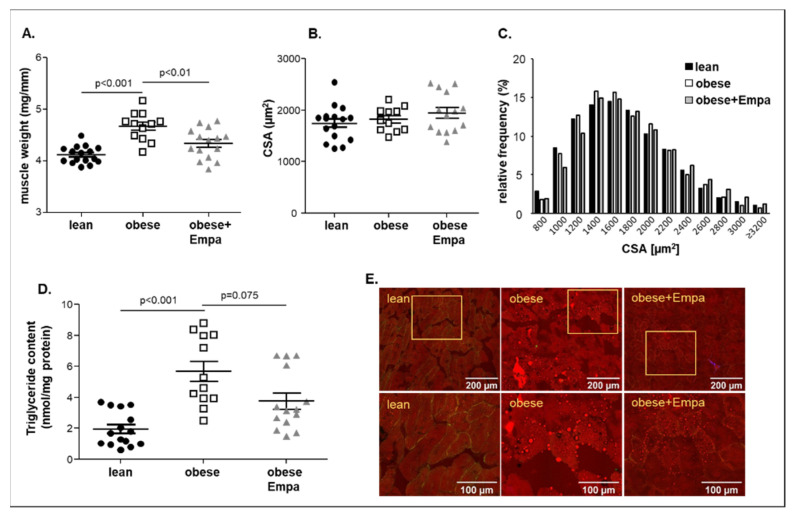
Muscle wet weight of the soleus muscle normalized to tibia length (**A**), cross-sectional area (CSA) (**B**,**C**) and triglyceride content (**D**) of the soleus muscle was measured in ZSF1-lean (lean, black circles), ZSF1-obese (obese, open squares) and ZSF1-obese rats treated with empagliflozin (obese + Empa, grey triangle) (*n* = 11–15 per group). Results are expressed as mean ± SEM. In addition, NLOM (nonlinear optical microscopy) pictures (**E**) to visualize fat deposition (red staining) in the quadriceps muscle of all three groups are shown.

Measuring soleus muscle weight and cross sectional area (CSA) at an age of 32 weeks, a significant increase in muscle weight (Figure 1A) with no change in CSA (Figure 1B,C) was evident in the obese animals when compared to the lean counterparts. This increase in muscle weight was also evident for the EDL (extensor digitorum longus) muscle (lean: 3.66 ± 0.04 mg/mm; obese: 3.99 ± 0.07 mg/mm; *p* < 0.001), whereas a reduction in muscle weight was observed for the TA (tibialis anterior) (lean: 14.93 ± 0.16 mg/mm; obese: 13.83 ± 0.18 mg/mm; *p* < 0.001).

This increase in muscle weight went along with an increased triglyceride content in the obese group (Figure 1D). The increased lipid deposition was located between the muscle fibers as well as inside the myocytes (Figure 1E). Feeding Empa to the ZSF1-obese animals resulted in a significant reduction in the soleus muscle weight (Figure 1A) with no significant change in CSA and a trend (*p* = 0.075) towards a reduction in lipid content (Figure 1C). Moreover, for the EDL muscle a significant reduction in muscle weight was detected (obese + Empa: 3.80 ± 0.05 mg/mm, *p* < 0.05 vs. obese). For the TA muscle, no change in muscle weight was seen after Empa treatment when compared to the obese untreated animals (obese + Empa: 13.83 ± 0.15 mg/mm). Screening the hematoxylin and eosin-stained muscle sections for myocytes showing centralized nuclei, as a marker for regenerating, no difference was observed between the three groups (lean: 1.86 cells with centralized nuclei per 1000 myocytes with peripheral nuclei; Obese: 2.12 cells with centralized nuclei per 1000 myocytes with peripheral nuclei; Obese + Empa: 2.00 cells with centralized nuclei per 1000 myocytes with peripheral nuclei). Assessment of capillary to fiber ratio (C:F) revealed a reduced C:F in the obese animals and Empa had no effect on C:F (lean: 2.60 ± 0.07; obese: 2.28 ± 0.08; obese + Empa: 2.26 ± 0.05; *p* < 0.05 lean vs. obese and obese + Empa).

**Figure 2:**
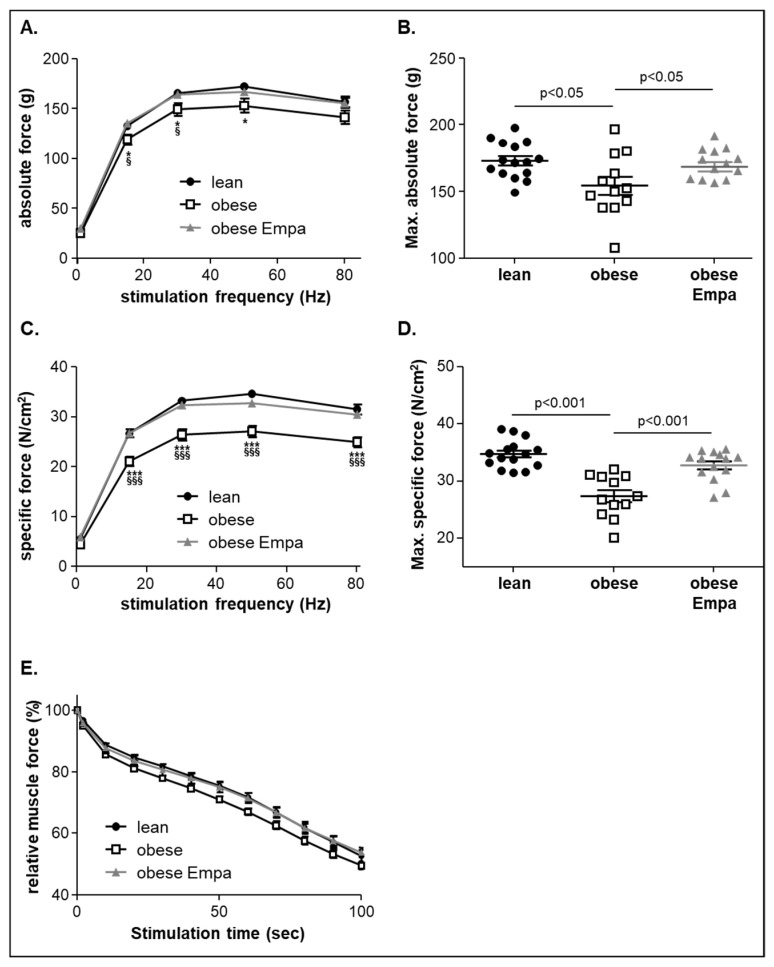
Absolute (**A**), maximal absolute force (**B**), specific (**C**) and maximal specific muscle force (**D**) was measured ex vivo from ZSF1-lean (lean, black circles), ZSF1-obese (obese, open squares) and ZSF1-obese rats treated with empagliflozin (obese + Empa, grey triangle) (*n* = 12–15 per group). Furthermore, muscle fatigue of the soleus muscle was assessed by repetitive stimuli in all three groups and expressed as relative muscle force (**E**). Results are expressed as mean ± SEM. * *p* < 0.05, *** *p* < 0.001 vs. lean; ^§^ *p* < 0.05, ^§§§^ *p* < 0.001 vs. obese + Empa.

Analyzing the skeletal muscle function of the soleus muscle ex vivo in an organ bath setting revealed a significant reduction in absolute (Figure 2A,B) and specific muscle force (Figure 2C,D) in the ZSF1-obese animals when compared to the ZSF1-lean control animals.

Feeding Empa to ZSF1-obese animals significantly improved maximal absolute (Figure 2B) and maximal specific muscle force (Figure 2D). With respect to muscle fatigue, a reduction in the ZSF1-obese animals was seen but this difference did not reach statistical significance. After Empa feeding, the values from the ZSF1-obese treated animals were not distinguishable from the ZSF1-lean controls (Figure 2E).

### 2.3. Impact of Empaliflozin on Muscle Atrophy Marker Expression

**Figure 3:**
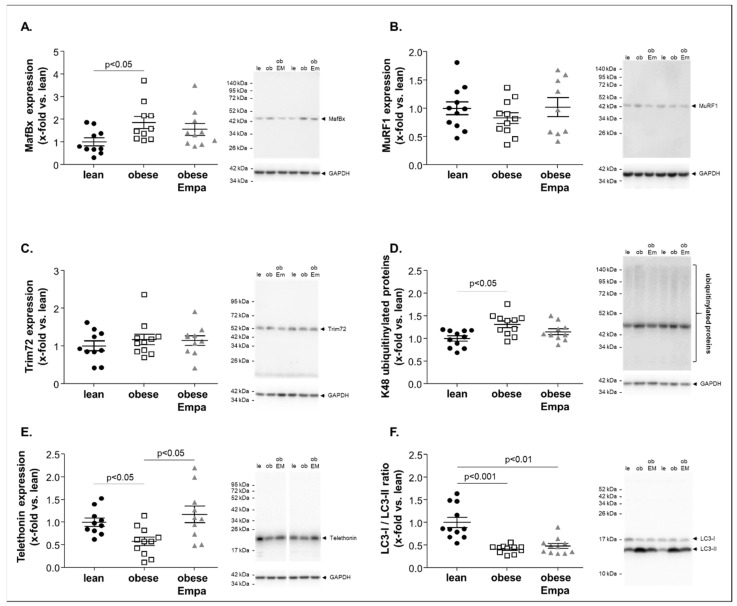
Protein expression of atrophy-related proteins (**A**–**F**) were quantified by western blot analysis in soleus muscle homogenates obtained from ZSF1-lean (lean, black circles), ZSF1-obese (obese, open squares) and ZSF1-obese rats treated with Empagliflozin (obese + Emp), grey triangle). As atrophy related proteins Muscle Atrophy F-Box (MAFBx) (**A**), muscle RING-finger protein-1 (MuRF1) (**B**), Tripartite Motif-containing Protein 72 (Trim72) (**C**), ubiquitinylated proteins (**D**), telethonin (**E**) and LC3B were measured. Results are expressed as mean ± SEM (*n* = 10–11 per group). Representative western blots are depicted.

To assess the molecular induction of muscle atrophy, the protein expression of selected proteins was quantified by western blot analyses. As shown in Figure 3A, the expression of MafBx (also known as atrogin 1) was significantly increased in the soleus muscle of ZSF1-obese animals when compared to the lean counterparts (Figure 3A). No significant upregulation was observed for two other ubiquitin E3 ligases, namely MuRF1 (Figure 3B) and Trim72 (Figure 3C). To explore if the increased expression of MafBx and other ubiquitin E3 ligases not investigated in the present study had any consequence with respect to the ubiquitin modification of proteins, we quantified the amount of ubiquitin-modified proteins. A specific focus was on ubiquitin moieties linked via K48, since this modification schedules the proteins for proteasome degradation [26]. As depicted in Figure 3D a significantly higher number of ubiquitin-modified proteins was evident in the soleus muscle of ZSF1-obese animals compared to lean. This increased expression of MafBx and the higher number of ubiquitin-modified proteins was associated with a significantly lower amount of telethonin (Figure 3E), a protein known to be degraded via the ubiquitin proteasome pathway [27]. In addition, an activation of autophagy (a reduced ratio of LC3-I/LC3-II) was evident (Figure 3F). After feeding Empa for 8 weeks, no significant difference of MafBx (Figure 3A) and ubiquitinylated proteins (Figure 3D) was observed when compared to lean whereas a significant higher expression of telethonin was detected when compared to untreated obese animals (Figure 3E). No impact of Empa on autophagy was observed (Figure 3F).

### 2.4. Impact of Empaliflozin on Mitochondrial Function

**Figure 4:**
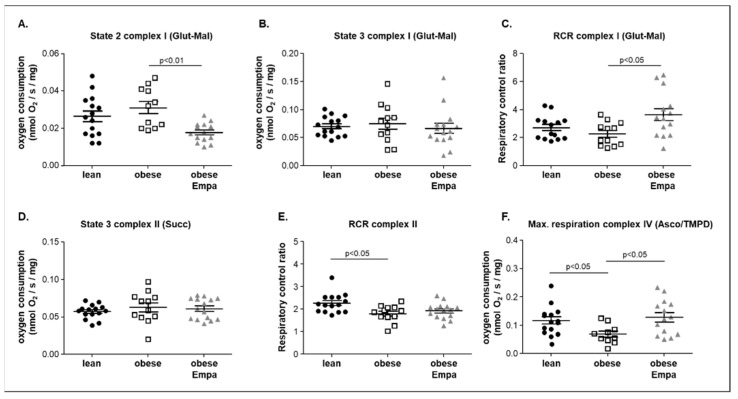
Mitochondrial function was assessed in saponin-skinned muscle fibers of the soleus muscle from ZSF1-lean (lean, black circles), ZSF1-obese (obese, open squares) and ZSF1-obese rats treated with Empagliflozin (obese + Empa, grey triangle) animals. To assess mitochondrial function complex I state 2 (**A**), state 3 (**B**) and the respiratory control ratio (RCR, (**C**)) using glutamine (Glut) and malate (Mal) as substrate was measured. For the assessment of complex II, succinate (Succ) was added to the saponin-skinned fibers and state 3 respiration (**D**) and the respiratory control ratio (RCR) (**E**) was quantified. To measure the maximal respiration of complex IV ascorbate (Asco) and the electron-donating compound, tetramethyl-p-phenylene diamine (TMPD) was added after the addition of the mitochondrial uncoupler FCCP (**F**). Results are expressed as mean ± SEM (*n* = 12–15 per group).

Using the saponin-skinned muscle fiber technology, mitochondrial function was assessed in the soleus muscle of all three groups. Quantifying basal respiration (only substrate added; state 2) a significant lower respiration rate was evident in the ZSF1-obese animals treated with Empa when compared to the untreated obese animals. No difference was documented between ZSF1-obese and ZSF1-lean animals (Figure 4A). Assessment of complex I state 3 ADP (state 3; glutamate/malate as substrate) showed that no difference was evident between the three groups (Figure 4B). Calculating the respiratory control ratio (RCR) for complex I, a significantly higher ratio was detected for the Empa-treated animals when compared to the untreated ZSF1-obese rats. (Figure 4C). After the addition of succinate and rotenone (state 3 of complex II), no significant difference was seen between the groups (Figure 4D). Calculating the RCR for complex II, a small but significant lower value was observed when comparing ZSF1-obese with ZSF1-lean animals (Figure 4E). This significant difference was no longer seen when comparing ZSF1-lean and ZSF1-obese Empa-treated animals. (Figure 4E). The most prominent effect on mitochondrial respiration was seen for the oxygen consumption of complex IV (Figure 4F). A significant lower oxygen consumption in the ZSF1-obese animals was observed when compared to the ZSF1-lean counterparts. Treating the ZSF1-obese animals for 8 weeks with Empa, a significantly higher value was detected in the obese treated animals compared to the obese untreated ones (Figure 4F). Correlating max. oxygen consumption of complex IV and maximal specific muscle force, a significant positive correlation between both parameters was noted (r = 0.45, *p* = 0.01).

**Figure 5:**
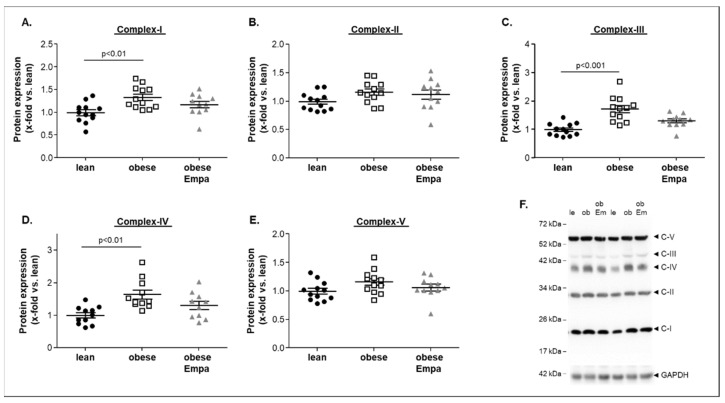
Protein expression of mitochondrial respiratory complex I–V (**A**–**E**) was quantified by western blot analysis in soleus muscle homogenates obtained from ZSF1-lean (lean, black circles), ZSF1-obese (obese, open squares) and ZSF1-obese rats treated with Empagliflozin (obese + Empa). A representative western blot is shown (**F**). Results are expressed as mean ± SEM (*n* = 11–12 per group).

To investigate if these alterations in mitochondrial function were related to changes in the protein expression of the different respiratory chain complexes, western blot analyses of the soleus muscle were performed. As shown in Figure 5A–E, a significantly higher protein expression of complex I (Figure 5A), complex III (Figure 5C) and complex IV (Figure 5D) was evident in the ZSF1-obese when compared to the ZSF1-lean group. Comparing the complex protein expression between the ZSF1-obese untreated and Empa-treated animals, no significant difference was noted (Figure 5A–E). A representative western blot is depicted in Figure 5F.

**Figure 6:**
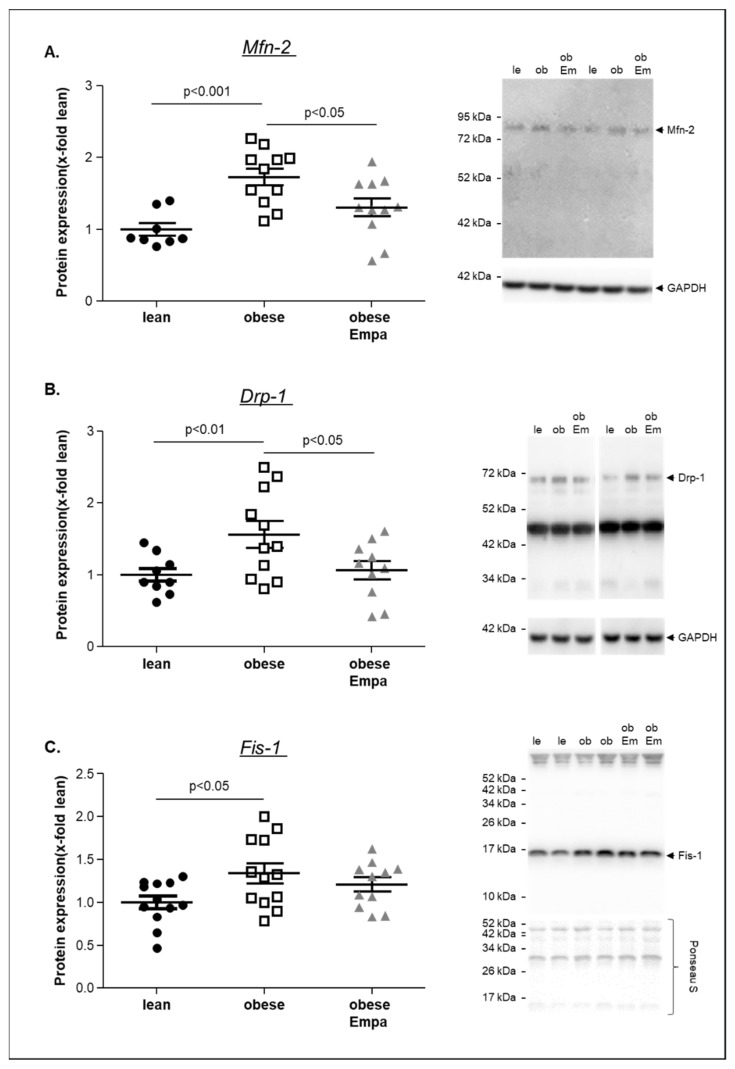
Protein expression of mitofusin-2 (Mfn-2) (**A**), Drp-1 (**B**) and Fis-1 (**C**) was quantified by western blot analysis in soleus muscle homogenates obtained from ZSF1-lean (lean, black circles), ZSF1-obese (obese, open squares) and ZSF1-obese rats treated with Empagliflozin (obese + Empa). Representative western blot is depicted. Results are expressed as mean ± SEM (*n* = 9–11 per group).

Furthermore, the protein expression of mitofusin-2 (Mfn-2), Drp-1 and Fis-1, markers for mitochondrial fusion and fission, was quantified in the soleus muscle (Figure 6A–C). For all markers, a significantly higher expression was evident in the obese animals and a significant reduction was seen in the Empa-treated animals for Mfn-2 and Drp-1 when compared to the untreated counterparts.

In summary, mitochondrial function seemed to be impaired in the ZSF1-obese animals, despite a higher protein expression of different complex proteins, and this impairment improved with Empa treatment (mainly seen in complex IV).

### 2.5. Impact of Empaliflozin on Metabolic Key Enzymes

**Figure 7:**
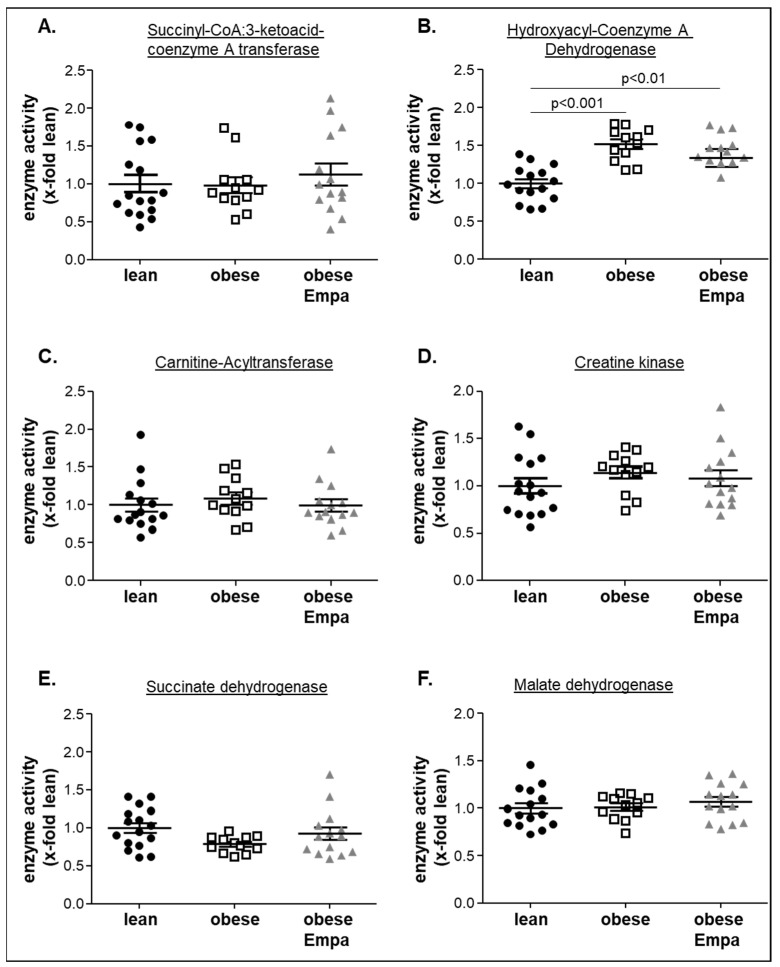
Enzyme activities of succinyl-CoA:3-ketoacid-coenzyme A transferase (**A**), hydroxyacyl-Coenzyme A dehydrogenase (**B**), carnitine-acyltransferase (**C**), creatine kinase (**D**), succinate dehydrogenase (**E**), and malate dehydrogenase (**F**) were measured in soleus muscle homogenates from ZSF1-lean (lean, black circles), ZSF1-obese (obese, open squares) and ZSF1-obese rats treated with Empagliflozin (obese + Empa). Results are expressed as mean ± SEM (*n* = 12–15 per group).

To analyze if alterations in different metabolic pathways were associated with alterations in muscle function, key enzymes of different metabolic pathways including ketone body utilization (succinyl-CoA:3-ketoacid-coenzyme A transferase, SCOT) (Figure 7A), ß-oxidation and transfer of fatty acids into the mitochondria (hydroxyacyl-Coenzyme A dehydrogenase (HOA-DH) and carnitine-acyltransferase (CPT)) (Figure 7B,C), energy transfer form mitochondria to the myofibers (creatine kinase) (Figure 7D) and Krebs cycle (succinate dehydrogenase and malate dehydrogenase) (Figure 7E,F) were analyzed. A significantly higher enzymatic activity was only seen for the HOA-DH in the obese and obese treated animals when compared to the lean counterparts (Figure 7B). For all the other metabolic key enzymes, no differences were observed between the three groups.

## 3. Discussion

Various skeletal muscle abnormalities are described in heart failure (HF) and they are closely associated with exercise intolerance. In particular, abnormal energy metabolism caused by mitochondrial dysfunction in skeletal muscle is a cause of decreased endurance exercise capacity. SGLT2i such as Empa and dapagliflozin have recently been reported to lower the risk of cardiovascular death in HFrEF and HFpEF and to increase exercise capacity (6 min walk test) in HFpEF after 12 weeks [28]. Mechanistically it is speculated that increased myocardial energy production and improved microvascular function are responsible for improved exercise capacity. The results of the present experimental study investigating the effect of 8 weeks of Empa treatment in an animal model of HFpEF with a specific focus on skeletal muscle can be summarized as follows:

- Improved skeletal muscle contractility and reduced lipid content with some alterations of atrophy-related proteins;
- Improved mitochondrial function especially in complex IV without modulating the protein expression of mitochondrial complex proteins;
- Had no effect on key enzymes of different metabolic pathways such as ketone body utilization, ß-oxidation or Krebs cycle.

In summary, the results of the present experimental study in HFpEF provide first line evidence that SKM function improves after Empa treatment and that these functional improvements are accompanied by better mitochondrial function, reduced myosteatosis and increased expression of telethonin. These amendments may partially explain the increase in exercise capacity seen in HFpEF patients after SGLT2i therapy.

### 3.1. Empagliflozin and Skeletal Muscle Mass and Function

There has been a concern that SGLT2i could induce skeletal muscle atrophy and sarcopenia due to the loss of carbohydrates, but the results presented in the current literature are inconsistent [29,30,31,32]. For example, treating type 2 diabetic patients with dapagliflozin either for 24 weeks [29] or 6 months [30] resulted in no significant change in skeletal muscle mass, whereas 4 weeks of luseogliflozin [32] or 24 weeks of ipragliflozin [31] reduced muscle mass significantly. The reason for this discrepancy is still unclear but we might hypothesize that different SGLT2i might have different effects on skeletal muscle mass. In the present study, investigating the effect of Empa on skeletal muscle mass and function in an animal model of HFpEF, we observed a reduction in muscle mass normalized to tibia length of the soleus muscle. Since no impact of Empa on the muscle cross-sectional area was observed, this loss of muscle weight was probably due to a reduced myosteatosis. This notion is supported by the observation that the triglyceride content of the soleus muscle was reduced by Empa. That Empa can reduce tissue triglyceride is in accordance with a recently published study in mice where the authors reported a significant reduction in liver triglyceride content after 4 weeks of Empa treatment [33]. A simple explanation for the lower triglyceride tissue content would be that lower blood glucose, as seen under Empa, stimulates lipolysis [34]. This would imply that we should observe a higher enzyme activity for enzymes of the ß-oxidation (i.e., hydroxyacyl-coenzyme A dehydrogenase) with Empa treatment. As documented in the present study, this is not the case, and therefore further studies are necessary to investigate the molecular effect leading to decreased tissue fat incorporation.

Impaired exercise capacity and reduced muscle function are hallmarks of HF, independent of HFrEF or HFpEF, which are closely associated with morbidity and mortality. Therefore, great efforts have been undertaken to increase muscle force and endurance in HF. One of the most efficient interventions is exercise training as proven in many clinical and experimental studies in HF. Since exercise training as interventional therapy is not always feasible (i.e., unmotivated patients, very cachectic patients), pharmaceutical therapies are urgently needed. With respect to HFpEF, only SGLT2i have proven beneficial effects with respect to hospitalization and mortality [14]. Data reporting an impact of Empa or other SGLT2i on skeletal muscle function are rare. The present study, performed in a validated HFpEF model [23,24,35], revealed a beneficial effect of Empa on absolute and specific muscle force with minor changes in muscle endurance. The impact of Empa on muscle endurance and force was also recently described in an experimental model of HFrEF. Nambu and colleagues [18] administered Empa for 4 weeks to mice 2 weeks post-myocardial infarction. Using in vivo measures for muscle endurance and strength, they documented that Empa ameliorated muscle endurance capacity without effecting cardiac function, food intake and skeletal muscle strength. In addition, mitochondrial oxidative phosphorylation with fatty acids as the substrate was increased. Feeding canagliflozin for 2 weeks to healthy C57BL/6J male mice also resulted in a small increase in grip strength without any change in muscle endurance or muscle mass [36]. What are the potential molecular mechanisms for this Empa-mediated skeletal muscle functional improvement? One explanation would be the improvement of mitochondrial oxidative phosphorylation (discussion see below) and the increased expression of telethonin due to a modulation of the ubiquitin proteasome system (see Figure 4). Telethonin anchors the N-terminal region of titin in the Z-disk [37] and as an adapter protein it links myofibrillar components with the membranous beta-subunit of the I(Ks) channel [38]. In addition, telethonin knockdown leads to embryonic paralysis, myocyte defects and sarcomeric disruption [39]. Therefore, it is conceivable that a modulation of telethonin expression will have an impact on muscle function. Another explanation would be that skeletal muscle functions improves with Empa due to a decrease in skeletal muscle fat infiltration (myosteatosis, see above). That myosteatosis is associated with reduced muscle function is clearly documented in the current literature (for review see [40]).

### 3.2. Empagliflozin and Mitochondrial Function

It is well recognized in the current literature that alterations in the skeletal muscle occur in HFpEF and that these alterations are partially responsible for exercise intolerance (for review see [41]). Besides structural and metabolic alterations, abnormalities in skeletal muscle oxidative phosphorylation and ATP generation have been reported [7,42,43]. These alterations are physiologically important, since ATP has to be replenished when a muscle contracts constantly. Our group was one of the first to report reduced mitochondrial respiratory capacity in the skeletal muscle of an animal model of HFpEF (Dahl salt-sensitive rat model) [7]. These experimental findings were recently supported by a clinical study in HFpEF patients showing impaired oxidative phosphorylation (measured indirect using the Creatine Chemical-Exchange Saturation Transfer (CrCEST) method) and reductions in proteins and complexes involved in energy fuel metabolism [44]. Impairment in oxidative phosphorylation (RCR value of complex II and respiratory activity of complex IV) was also confirmed in the present study measuring oxygen consumption in saponin-skinned muscle fibers. These functional impairments were not accompanied by a reduced expression of the respective mitochondrial complexes, but an even higher expression of complexes I, III and IV was evident. This can be interpreted as a compensatory mechanism of the mitochondria to counteract the functional impairment. The treatment with Empa for 8 weeks showed beneficial effects on the RCR of complex I and maximal respiration rates of complex IV. To our knowledge, this is the first report documenting the positive effects on mitochondrial function in the skeletal muscle of HFpEF. The importance of modulating mitochondrial respiration rates for muscle contractility is furthermore supported by the positive significant correlation between complex IV respiration rates and maximal specific muscle force. Our results with respect to the impact of Empa on mitochondrial function are in accordance with a recently published study in HFrEF [18]. Nambu and colleagues investigated in a post-myocardial infarction (MI) model the effect of 4 weeks of Empa on skeletal muscle and reported that Empa ameliorated the decrease in mitochondrial oxidative phosphorylation capacity with fatty acid substrates [18]. What are the potential molecular mechanisms mediating the beneficial effect of Empa on mitochondrial function in the skeletal muscle? This answer can only be speculative since no data are available up to now for skeletal muscle myocytes. Recently Cai and colleagues [45] provided experimental evidence in an ischemia/reperfusion model that Empa preserves mitochondrial function by activating the AMPK1α/ULK1/FUNDC1 mitophagy pathway and reducing mitochondrial apoptosis in cardiac microvascular endothelial cells. The modulatory effect of Empa on mitochondrial size was also confirmed in cardiomyocytes after myocardial infarction [46]. Moreover, in the present study, we have some evidence that Empa modulates mitochondrial fusion and fission by altering the expression of Drp1, Mfn2 and Fis-1. The upregulation of Drp1 and Mfn2 in heart failure is in accordance with our previous study analyzing human diaphragm samples from HF patients [47]. Nevertheless, further studies are necessary to explore the molecular mechanism finally leading to the beneficial effects of Empa on skeletal muscle function.

### 3.3. Empagliflozin and Metabolic Alterations

It has been hypothesized that SGLT2i prevent heart failure through improving ATP generation from ketone body and fatty acid oxidation [48,49]. Nevertheless, this hypothesis is considered controversial [50] because inconsistent results in animal models have been reported. For example, increased serum level of ß-hydroxybutyrate were reported after feeding Empa to HFrEF animals (LAD ligation) [18], whereas the treatment of db/db mice with Empa did not improve cardiac ketone oxidation rates [51]. Moreover, in the present study analyzing the impact of Empa on skeletal muscle function and metabolic alteration, no difference in key enzyme activities for ß-oxidation, ketone metabolism and TCA was observed between the three groups. One explanation for this discrepancy may be the severity of disease treated with Empa (HFrEF vs. HFpEF). Nevertheless, more investigations are clearly needed to prove that Empa impacts on myocardial and skeletal muscle function by modulating substrate metabolism and fueling mitochondrial ATP generation.

### 3.4. Study Limitations

The present study is the first report on the beneficial effects of Empa on skeletal muscle function in HFpEF mediated by improving mitochondrial function via the modulation of mitochondrial dynamics and the reduction in myosteatosis. However, the following limitations have to be considered.

First, in the present study, no measurements of exercise performance—running test or cardiopulmonary assessment—were performed to directly assess exercise capacity. This might be important since exercise capacity is not only determined by skeletal muscle force but also by other factors such as the capillarization of the muscle and the ability of the myocytes to extract oxygen from the blood. These are all factors known to be altered in HFpEF [52,53,54].

Second, we only analyzed the functional and molecular parameters of the soleus muscle, a slow twitch muscle with high endurance, and omitted fast twitch (EDL or TA muscle) or mixed muscle types (quadriceps muscle). In an earlier study analyzing the impact of a MuRF1 inhibitor [25] in the same animal model as used in the present study we detected a lower contractility of the soleus and EDL muscle. These results at least suggest that both muscle types are functionally impaired in HFpEF and show beneficial effects towards treatment. Therefore, we may assume that also in the present study the EDL may show beneficial effects towards Empa treatment.

Third, no histological analyses such as electron microscopy were performed to assess mitochondrial morphology and distribution inside the muscle cell. Electron microscopy would provide critical information about mitochondrial content, cristae density, organization, formation of autophagosomes, and any other abnormalities commonly observed in various disease conditions such as heart failure. In a recently published study [47] we demonstrated the accumulation of intermyofibrillar and subsarcolemmal mitochondria with a predominately small size in the diaphragm of heart failure patients.

Forth, the study was only performed in female rats. Therefore, the transferability of the present results to the total HFpEF population, also including males, needs to be confirmed. Nevertheless, it should be noted that data from registry and community-based studies demonstrate that patients with HFpEF are more often older, female, and more likely to have multiple comorbidities, including hypertension, diabetes, pulmonary disease, chronic kidney disease, and obesity [55,56].

## 4. Materials and Methods

### 4.1. Study Design

**Figure 8:**
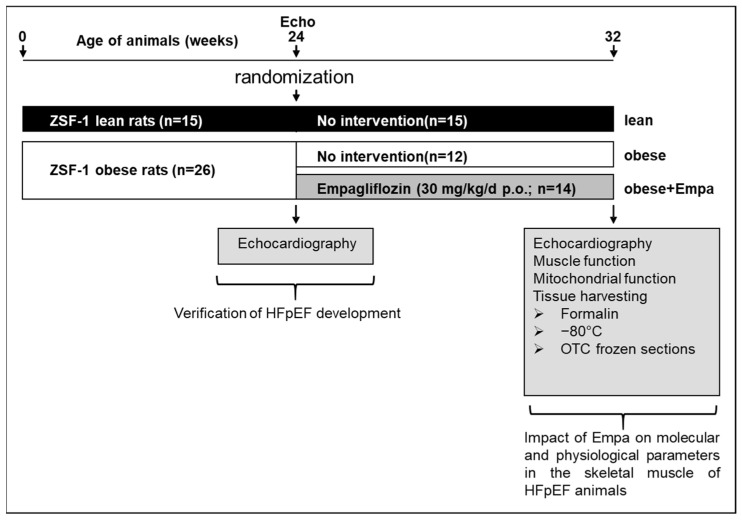
Study design. A schematic drawing of the study design is depicted. ZSF1-lean (*n* = 15) and ZSF1-obese (*n* = 26) were included into the study. At the age of 24 weeks, after the development of signs of HFpEF, ZSF1-obese animals were randomized into two groups either receiving no intervention (*n* = 12) or Empa (30 mg/kg/d p.o.) for 8 weeks. At the end of the study echocardiography (Echo) was performed and skeletal muscle tissue was harvested for physiological and molecular analyses. Echo = echocardiography.

ZSF1-lean (*n* = 15) and ZSF1-obese (*n* = 26) female animals were purchased from Charles River. At the age of 24 weeks, echocardiography was performed to document the development of HFpEF in the ZSF1-obese animals. Thereafter, the ZSF1-obese animals were randomized either into a placebo group (normal chow and water ad libidum, obese, *n* = 12) or a treatment group (*n* = 14) receiving Empa (30 mg/kg/d p.o., obese + Empa). ZSF1-lean animals served as healthy control group (lean, *n* = 15). After 8 weeks of treatment, echocardiography was performed and the animals were sacrificed by bilateral thoracotomy while the rats were under a surgical plane of anesthesia (Figure 8).

The right soleus muscle (SO) was prepared for functional measurements, whereas a small piece of the left SO was used for preparation of skinned muscle fibers for the assessment of mitochondrial respiration. In addition, SKM weights (SO, extensor digitorum longum (EDL) and tibialis anterior (TA)), were determined and snap frozen in liquid nitrogen. All experiments and procedures were approved by the local animal research council, TU Dresden and the Landesbehörde Sachsen (TVV 34/2020).

### 4.2. Echocardiography and Invasive Hemodynamic Measurements

Rats were anesthetized by isoflurane (1.5–2%) and transthoracic echocardiography was performed using a Vevo 3100 system and a 21-MHz transducer (Visual Sonic, Fujifilm) to assess cardiac function as recently described [23,24]. For systolic function B- and M-Mode of parasternal long- and short axis were measured at the level of the papillary muscles. Diastolic function was assessed in the apical 4-chamber view using pulse wave Doppler (for measurement of early (E) and atrial (A) waves of the mitral valve velocity) and tissue Doppler (for measurement of myocardial velocity (é)) at the level of the basal septal segment in the septal wall of the left ventricle. Functional parameters (i.e., LV ejection fraction (LVEF) and stroke volume (SV) and E/é ratio) were obtained using the Vevo LAB software (version 3.1.1, VisualSonics Inc, Amsterdam, Netherlands).

Prior to organ harvest, invasive hemodynamic measurements were performed as recently described [24] in the left ventricle (LV) of anaesthetized (i.m. injection of ketamine (105 mg/kg) and xylazine (7 mg/kg)) but spontaneously breathing rats. For LV pressure-volume measurement the right carotid artery was cannulated with a Rat PV catheter (SPR-838, ADInstruments Limited, Oxford, UK), which was gently placed in the middle of the left ventricle.

### 4.3. Skeletal Muscle Function and Cross Sectional Area

The right soleus was dissected and mounted vertically in a Krebs–Henseleit buffer-filled organ bath between a hook and force transducer, with the output continuously recorded and digitized (1205A: Isolated Muscle System—Rat, Aurora Scientific Inc., Aurora, ON, Canada). Ex vivo muscle function was assessed by platinum electrodes stimulating the muscle with a supra-maximal current (700 mA, 500 ms train duration, 0.25 ms pulse width) from a high-power bipolar stimulator (701C; Aurora Scientific Inc., Aurora, ON, Canada). The muscle bundle was set at an optimal length (Lo) equivalent to the maximal twitch force produced. A force-frequency protocol was then performed at 1, 15, 30, 50, and 80 Hz, separated by 1-min rest intervals.

After a 5-min period in which muscle length was measured using a digital micrometer, the muscle underwent a fatigue protocol over 5 min (40 Hz every 2 s with a 500-ms train duration). The muscle was subsequently detached, trimmed free from fat and tendon, blotted dry on filter paper, and weighted. Muscle force (N) was normalized to muscle cross-sectional area (cm^2^) by dividing muscle mass (g) by the product of optimal length (cm) and estimated muscle density (1.06 g/cm^3^), which allowed specific force (in N/cm^2^) to be calculated [7,23,24].

### 4.4. Muscle Mitochondrial Respiration

The respiratory parameters of the total mitochondrial population were studied in saponin-skinned fibers. Respiratory rates were determined by using a Clark electrode (Strathkelvin Instruments, Motherwell, UK) in an oxygraphic cell at 25 °C with continuous stirring. To avoid oxygen diffusion limitation, oxygen concentration was increased to ~400 μmol/L by adding pure oxygen and was kept above 270 μmol/L throughout the experiment.

Soleus muscle fibers were isolated in permeabilization solution (SolP) containing (in mmol/L): 2.77 CaK2EGTA, 7.23 K2EGTA, 6.56 MgCl_2_, 5.7 Na2ATP, 15 phosphocreatine (PCr), 20 taurine, 0.5 DTT, 50 K methane-sulfonate, 20 imidazole (pH 7.1) and incubated for 30 min in SolP with 50 μg/mL saponin. Permeabilized fibers were transferred to respiration solution (SolR) (in mmol/L: 20 taurine, 20 HEPES, 10 KH_2_PO_4_, 0.5 EGTA, 3 MgCl_2_, 0.11 sucrose, 60 K-lactobionate (pH 7.4)) for 10 min to wash out adenine nucleotides and PCr. All steps were carried out at 4 °C with continuous stirring. Respiration rates of 1–5 mg of skinned fibers were measured at 25 °C in 1 mL of SolR that contained 1 mg/mL bovine serum albumin. The following substrates were added sequentially and oxygen consumption was monitored: (i) glutamate (10 mmol/L), malate (2.0 mmol/L), (complex I state 2 respiration); (ii) adenosine diphosphate (5.0 mmol/L; measure of complex I oxidative phosphorylation); (iii) cytochrome C (10 μmol/L; test for membrane integrity); (iv) succinate (10 mmol/L; oxidative phosphorylation of complex I + II); (v) rotenone (0.5 mmol/L; oxidative phosphorylation of complex II); (vi) FCCP (0.5 μmol/L, maximal uncoupled complex II respiration); (vii) antimycin A (2.5 μmol/L, as a complex III inhibitor); (viii) ascobate/N,N,N′,N′-tetramethyl-p-phenylenediamine dichloride (2 mmol/L/0.5 mmol/L, maximal uncoupled complex IV respiration). After the experiment, fiber bundles were blotted dry and weighted. Rates of respiration are given in nmoles O_2_ per second per mg wet weight.

### 4.5. Western Blot Analysis

For western blot analyses, frozen soleus muscle was homogenized in Relax buffer (90 mmol/L HEPES, 126 mmol/L potassium chloride, 36 mmol/L sodium chloride, 1 mmol/L magnesium chloride, 50 mmol/L EGTA, 8 mmol/L ATP, 10 mmol/L creatine phosphate, pH 7.4) containing a protease inhibitor mix (Inhibitor mix M, Serva, Heidelberg, Germany) and sonicated. Protein concentration was determined (BCA assay, Pierce, Bonn, Germany) and aliquots (5–20 μg) were separated by SDS-polyacrylamide gel electrophoresis. Proteins were transferred to a polyvinylidene fluoride membrane (PVDF) and incubated overnight at 4 °C using the following primary antibodies: MafBx, MuRF1, Trim72, anti-ubiquitin linkage-specific K48, Mfn2 and telethonin (all 1/1000, Abcam, Cambridge, UK), LC3B (1:1000, Sigma, Deisenhofen, Germany), total OXPHOS components (1:250, Abcam, Cambridge, UK), Fis1 and DRP1 (1:1000, Proteintech, Planegg-Martinsried, Germany). Membranes were subsequently incubated with a horseradish peroxidase-conjugated secondary antibody and specific bands visualized by enzymatic chemiluminescence (Super Signal West Pico, Thermo Fisher Scientific Inc., Bonn, Germany) and densitometry quantified using a 1D scan software package (Scanalytics Inc., Rockville, MD, USA). Measurements were normalized to the loading control GAPDH (1/30,000; HyTest Ltd., Turku, Finland) or to overall protein loading as determined by Ponceau S staining. All data are presented as x-fold change relative to lean.

### 4.6. Enzyme Activity Measurements

Soleus tissue was homogenized in a Relax buffer and aliquots were used for enzyme activity measurements. Enzyme activities for succinate dehydrogenase (SDH, EC 1.3.5.1), ß-hydroxyacyl-COA dehydrogenase (EC 1.1.1.35), malate dehydrogenase (EC 1.1.1.37), creatine kinase (EC 2.7.3.2), carnitine-acyltransferase (EC 2.3.1.21) and succinyl-CoA:3-ketoacid CoA transferase (EC 2.8.3.5) were measured spectrophotometrically as previously described in detail [57,58,59,60]. Enzyme activity data are presented as the x-fold change relative to lean.

### 4.7. Histological Analysis

In preparation for nonlinear optical microscopy (NLOM), a nondestructive and label-free technique based on multiphoton processes [61], freshly harvested quadriceps muscles were fixed in 4% paraformaldehyde overnight followed by a 24-h-incubation in sucrose solution (20%) before cryo-embedding (Tissue-Tek^®^ O.C.T.™ compound, Sakura Finetek Germany GmbH, Umkirch, Germany). Cryo-embedded skeletal muscle sections (14 µm) were placed on glass slides, kept moist by a drop of phosphate buffered saline, and natively used for NLOM which was performed using laser excitation provided by two picosecond near-infrared Erbium fiber sources. Technical details were previously described [62]. Three signals were simultaneously acquired and combined in one RGB image: coherent anti-Stokes Raman scattering (CARS) in the red channel, two-photon excited fluorescence (TPEF) in the green channel and second harmonic generation (SHG) in the blue channel. For the identification of lipid structures, we applied CARS [63] and SHG for the detection of highly ordered structures lacking inversion symmetry such as fibrillary collagen and the combination of SHG and TPEF for myosin [64].

For the assessment of muscle cross-sectional area (CSA), paraffin-embedded soleus muscle was sectioned (4 µm), mounted on glass slides and stained with hematoxylin and eosin. The fiber cross-sectional area (CSA) was evaluated by imaging software (Zen imaging software, Zeiss, Jena, Germany). A minimum of 200 fibers per section were measured.

To assess the capillary to fiber ratio, paraffin-embedded soleus muscle was sectioned (4 µm), mounted on glass slides and incubated with a specific antibody against aquaporin-1 (Chemicon AB3065, 1:100 diluted, O/N 4 °C) followed by a peroxidase-coupled anti rabbit antibody (Dako, Hamburg). AEC was used as peroxidase stain and the sections were imaged at magnification × 40 (Revolve microscope, Echo Laboratories Inc., San Diego, CA, USA). The number of fibers and capillaries were counted in a defined area and the capillary to fiber ratio (F:R) was calculated.

### 4.8. Triglyceride and Glucose Content

Triglyceride content in the soleus muscle was measured using a triglyceride quantification kit (Sigma, MAK266) according to the manufacturer’s protocol. Glucose concentration in serum and urine was measured using a specific quantification kit (Sigma, MAK013) according to manufacturer’s protocol.

### 4.9. Statistical Analyses

Data are presented as mean ± SEM. In the case the data are normally distributed One-way analysis of variance (ANOVA) followed by Bonferroni post hoc was used to compare groups, while the Kruskal-Wallis test followed by Dunn’s post hoc test was used for not normally distributed data. Two-way repeated measures ANOVA followed by Bonferroni post hoc was used to assess contractile function (GraphPad Prism). Significance was accepted as *p* < 0.05.

## 5. Conclusions and Future Directions

**Figure 9:**
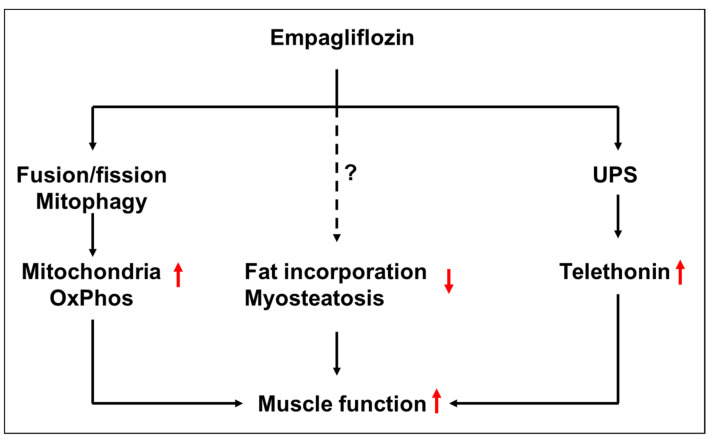
Hypothetical working model how Empagliflozin might improve skeletal muscle function in settings of HFpEF by the modulation of mitochondrial oxidative phosphorylation, myosteatosis and telethonin expression. UPS = ubiquitin proteasome system.↑, ↓ = up- or downregulated by Empa, ? and dottet line = exact pathway not defined yet.

Empa administered over 8 weeks to an animal model of HFpEF improved skeletal muscle function when compared to untreated animals. These beneficial effects were partially mediated by an improvement in mitochondrial function, a reduction in myosteatosis and an increase in the expression of telethonin, a protein involved in anchoring titin to the Z-disk (Figure 9). These alterations in the peripheral skeletal muscle elicited by Empa may contribute to improving exercise performance in HFpEF patients [28]. Nevertheless, further studies are necessary to investigate the precise molecular mechanisms responsible for the beneficial effects of Empa. In addition, studies investigating the crosstalk between the SKM and the heart are warranted.

## Figures and Tables

**Table 1:** Animal characteristics at 32 weeks of age.

Parameter	Lean (*n* = 15)	Obese (*n* = 12)	Obese + Empa (*n* = 14)
Body weight (g)	255 ± 4	532 ± 9 ***	488 ± 6 ***^,§§§^
Tibia length (TL, mm)	35.6 ± 0.1	35.6 ± 0.1	35.3 ± 0.1
Heart weight/TL (mg/mm)	27.75 ± 0.65	43.82 ± 2.02 ***	36.70 ± 1.05 ***^,§§§^
Lung weight (wet/dry)	4.56 ± 0.02	4.22 ± 0.04 ***	4.22 ± 0.06 ***
Lung wet weight/TL (mg/mm)	10.89 ± 0.11	11.91 ± 0.21 ***	11.04 ± 0.18 ^§§^
Kidney weight/TL (mg/mm)	28.2 ± 0.5	46.6 ± 1.4 ***	49.4 ± 1.0 ***
blood glucose (mmol/L)	19.9 ± 1.8	37.2 ± 1.5 ***	25.6 ± 0.6 ^§§§^
Urinary glucose (mmol/L)	2.3 ± 0.6	15.2 ± 3.8	142.5 ± 21.6 ***^,§§§^
**Echocardiography/invasive hemodynamic**			
LVEF (%)	68.0 ± 1.4	67.7 ± 1.7	68.0 ± 1.2
LVFS (%)	23.6 ± 1.1	24.9 ± 1.1	22.9 ± 0.8
Aortic blood pressure (mmHg)	97 ± 3	129 ± 3 ***	117 ± 5 ***
E/é	17.5 ± 0.7	22.8 ± 1.1 ***	19.5 ± 0.8 ^§^
LVEDP (mmHg)	5.96 ± 0.35	8.02 ± 0.61 ***	7.05 ± 0.37 ^§^

TL = tibia length; LVEF = left ventricular ejection fraction; LVFS = left ventricular shortening fraction; LVEDP = left ventricular end-diastolic pressure. *** *p* < 0.001 vs. lean; ^§^ *p* < 0.05, ^§§^ *p* < 0.01, ^§§§^ *p* < 0.001 vs. obese.

## Data Availability

Not applicable.
